# Neurobehavioral Consequences of the 5′-AMP-Induced Torpor-like Hypothermic State

**DOI:** 10.3390/life16091561

**Published:** 2026-09-17

**Authors:** Alessandro Stronati, Giorgia Macchioni, Arianna Racca, Daniela Santucci, Matteo Cerri

**Affiliations:** 1 Department of Intensive Care Medicine and Anesthesiology, Università Cattolica del Sacro Cuore, 00168 Rome, Italy; alestronati7@gmail.com; 2 Center for Behavioral Sciences and Mental Health, Istituto Superiore di Sanità, Viale Regina Elena 299, 00161 Rome, Italy; 3 Department of Biomedical and Neuromotor Sciences, Alma Mater Studiorum, Università di Bologna, 40126 Bologna, Italy

**Keywords:** torpor-like hypothermic state, 5′-AMP, synthetic torpor, hibernation, space flight, suspended animation, pharmacological torpor, hypometabolic state

## Abstract

Torpor is a hypometabolic and hypothermic state that enables survival under extreme energetic constraints. Pharmacological induction with 5′-AMP produces a controllable torpor-like hypothermic state with hypometabolic features documented for this model, relevant to long-duration spaceflight. Such a state could mitigate the physiological hazards of deep-space missions, including cosmic radiation exposure and muscle wasting, and, by minimizing oxygen demand, preserve neural integrity and enhance radiation tolerance. However, translational application requires confirmation that induced hypometabolism does not impair cognitive or behavioral function; this is especially pressing given evidence that the state causes reversible tau hyperphosphorylation at phospho-sites also altered in Alzheimer’s disease, but without tau aggregation. We subjected C57BL/6 mice to 5′-AMP combined with a mildly reduced ambient temperature (15–18 °C) and compared them with cold-exposed and room-temperature vehicle controls using systematic neurobehavioral testing. Despite the deep hypothermia (and the hypometabolism documented for this model), Y-maze spontaneous alternation performance was unaffected. Locomotor activity fell transiently during post-arousal recovery, with concurrent increases in self-directed behaviors. These results support further evaluation of this reversible metabolic intervention for critical care medicine and future human space missions.

## 1. Introduction

Torpor is a reversible state of deep metabolic depression characterized by reductions in body temperature, heart rate, respiratory rate, and overall energy expenditure [1,2,3]. Across mammalian hibernators, it confers survival advantages under limited resources and entails systemic physiological reprogramming, with ultrastructural and cellular modifications spanning multiple organs [4,5,6].

Recent advances have begun to unravel the central circuit that controls torpor, and these findings have fueled efforts to identify methods that mimic its clinically useful features (a condition termed synthetic torpor). We use this term to align with the literature concept, not as a claim that any given protocol reproduces natural torpor. These approaches use pharmacological, multidrug, or neuromodulatory interventions [7,8,9,10,11,12,13,14,15] that converge on preoptic, dorsomedial hypothalamic, and raphe pallidus circuits known to coordinate hypometabolic responses [16,17].

While the physiological features reported for synthetic torpor models resemble natural torpor, their behavioral and neurological consequences remain poorly characterized. Natural torpor entails not only a profound reduction in metabolic rate but also distinct changes in neural function, thermoregulatory reflexes, posture, and locomotion [16,18]. Whether pharmacological induction reproduces these aspects, or instead introduces unintended effects, remains unclear [18,19,20]. Of particular concern has been the reversible hyperphosphorylation of tau, abnormal forms of which are a defining feature of Alzheimer’s disease, which has been observed during both natural torpor and synthetic torpor [20]. In torpid mammals, this phosphorylation is fully reversible and spares the tangle-associated microtubule-binding-region tau fragments [21,22], indicating a regulated response rather than a pathological one; whether it nonetheless carries functional consequences remains untested.

Systemic 5′-AMP induces a reversible hypothermic state in mice that prior studies have characterized as hypometabolic [23,24,25]. We therefore describe this 5′-AMP-induced state as a torpor-like hypothermic state, or equivalently a synthetic torpor-like hypothermic state, rather than synthetic torpor. Because metabolic rate was not measured in the present study, the term denotes the induction protocol and its previously documented metabolic phenotype rather than independently verified metabolic suppression in this cohort, and whether the metabolic decline is actively regulated or partly a passive consequence of cooling remains debated [26,27,28,29]. Indirect calorimetry in this model shows that oxygen consumption falls in two phases: an initial rapid decline that is independent of body and ambient temperature [23], followed by a decrease in total energy expenditure alongside a behavioral preference for cooler environments, indicating a lowered thermoregulatory set point [24].

This metabolic effect is independent of the adenosine A1 and A3 receptors [24] and is accompanied by reduced mitochondrial respiration and oxygen demand [25], which has been interpreted as active metabolic suppression rather than purely passive cooling. Here, we investigate the behavioral effects of the torpor-like hypothermic state, induced in mice by combining 5′-AMP with a mildly reduced ambient temperature (15–18 °C). Mice enter daily torpor spontaneously when food is withheld at a cool ambient temperature [30,31], and 5′-AMP reproduces this state on demand under the same conditions [23,24,32], making it a practical model. By quantifying post-arousal behaviors (Table 1), we test whether the torpor-like hypothermic state preserves basic cognitive and motor functions. In natural torpor, the pronounced neuronal and synaptic remodeling of the bout, including reversible tau hyperphosphorylation and transient dendritic spine regression, is largely restored soon after arousal under suitable conditions [21,33,34]. An analogous reversible tau hyperphosphorylation is induced by synthetic torpor in rats [20], and neuronal electrical and metabolic activity recovers regionally on rewarming [3]. Behavioral outcomes are nonetheless task- and species-specific, ranging from preserved hippocampal-dependent memory in hibernating golden hamsters [34] and largely retained spatial memory in golden-mantled ground squirrels [35] to impaired retention of learned tasks in European ground squirrels [36]. Establishing whether the torpor-like hypothermic state reproduces this generally reversible but mixed profile is important in evaluating its safety for translational applications.

Controlled hypometabolism has been proposed as a countermeasure for the hazards of long-duration space missions, including radiation exposure, muscle atrophy, bone demineralization, and the psychological burden of prolonged confinement, and as a means of extending intervention windows in time-sensitive clinical care and protecting healthy tissue during radiotherapy [17,37,38,39,40,41]. Both prospects presuppose a behavioral and neurological safety profile that has not yet been established.

## 2. Results

### 2.1. Temperature Monitoring During the Torpor-like Hypothermic State

**Figure 1:**
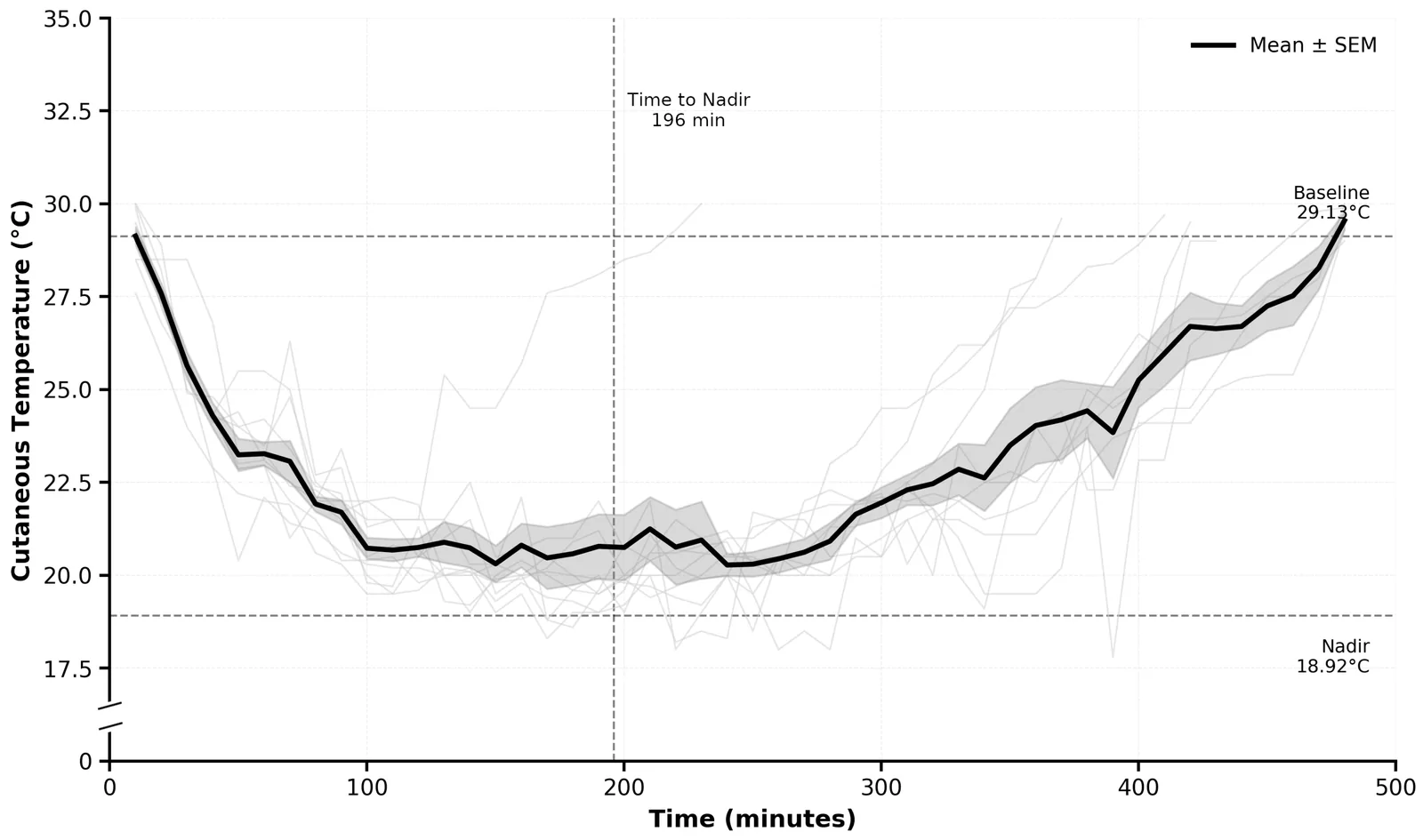
Temperature dynamics during the 5′-AMP-induced torpor-like hypothermic state in mice. Cutaneous temperature was recorded every 10 min with a non-contact infrared thermometer (ERICKHILL 600 C) following intraperitoneal injection of 5′-AMP (400 mg/kg) in female C57BL/6 mice (*n* = 10) housed at 15 to 18 °C. Light gray lines trace individual animals; the black line and gray shading show the population mean ± SEM. Dashed horizontal lines mark the baseline (29.13 ± 0.24 °C) and lowest recorded temperature (18.92 ± 0.34 °C); the vertical dashed line marks the time to temperature minimum (196.0 ± 26.9 min). The low coefficient of variation across individual temperature minima (5.4%) indicates consistent cutaneous hypothermic depth. Because skin and core temperature can diverge during peripheral vasoconstriction, this is not a measure of torpor depth (see Limitations). Cold-exposed control animals (Saline and Cold Exposure) maintained a mean cutaneous temperature of 28.3 °C (95% CI 27.7 to 28.8 °C) across the cold-exposure period, with no torpor-like decline.

The torpor-like hypothermic state was induced by combining intraperitoneal 5′-AMP (400 mg/kg) with housing at a mildly reduced ambient temperature (15–18 °C). To separate the effects of 5′-AMP from those of cold alone, the two vehicle-treated control groups received either the same cold exposure (Saline and Cold Exposure, 15–18 °C) or room-temperature housing (Saline, 22 °C). Cold exposure was therefore shared between the 5′-AMP and Cold Exposure group and the Saline and Cold Exposure group, with only 5′-AMP distinguishing them. Temperature recordings from all treated animals in the 5′-AMP and Cold Exposure group (*n* = 10) are shown in Figure 1. Administration of 5′-AMP reliably induced a deep hypothermia in every animal. Cutaneous surface temperature fell from a baseline of 29.13 ± 0.24 °C to a nadir of 18.92 ± 0.34 °C, with a mean drop of 10.21 ± 0.51 °C (paired *t*-test: t(9) = 20.04, *p* < 0.001). The depth of cutaneous hypothermia was highly consistent across animals: individual lowest temperatures ranged from 17.80 to 21.30 °C, with a coefficient of variation of only 5.4%. The timing of the response, by contrast, varied between individuals: the temperature nadir was reached at 196.0 ± 26.9 min post-injection (range: 100–390 min), after which all animals rewarmed spontaneously without external heating and returned to normothermia (>=28 °C) at 397.0 ± 26.8 min post-injection (range: 190–480 min).

Neither control group entered torpor. In the cold-exposed controls (Saline and Cold Exposure, 15 to 18 °C), cutaneous temperature sampled in three animals (the first three cold-exposed controls, enrolled across the first three experimental days) averaged 28.3 °C (95% CI 27.7 to 28.8 °C), with no torpor-like decline at any sampled time point and values far above the 5′-AMP and Cold Exposure nadir of 18.92 °C; the room-temperature controls (Saline, 22 °C) remained normothermic. This is the expected response, since fed C57BL/6 mice defend normothermia at sub-thermoneutral temperatures and enter daily torpor only under caloric restriction [42].

### 2.2. Behavioral Observations

#### Locomotor Activity and Crossings

**Figure 2:**
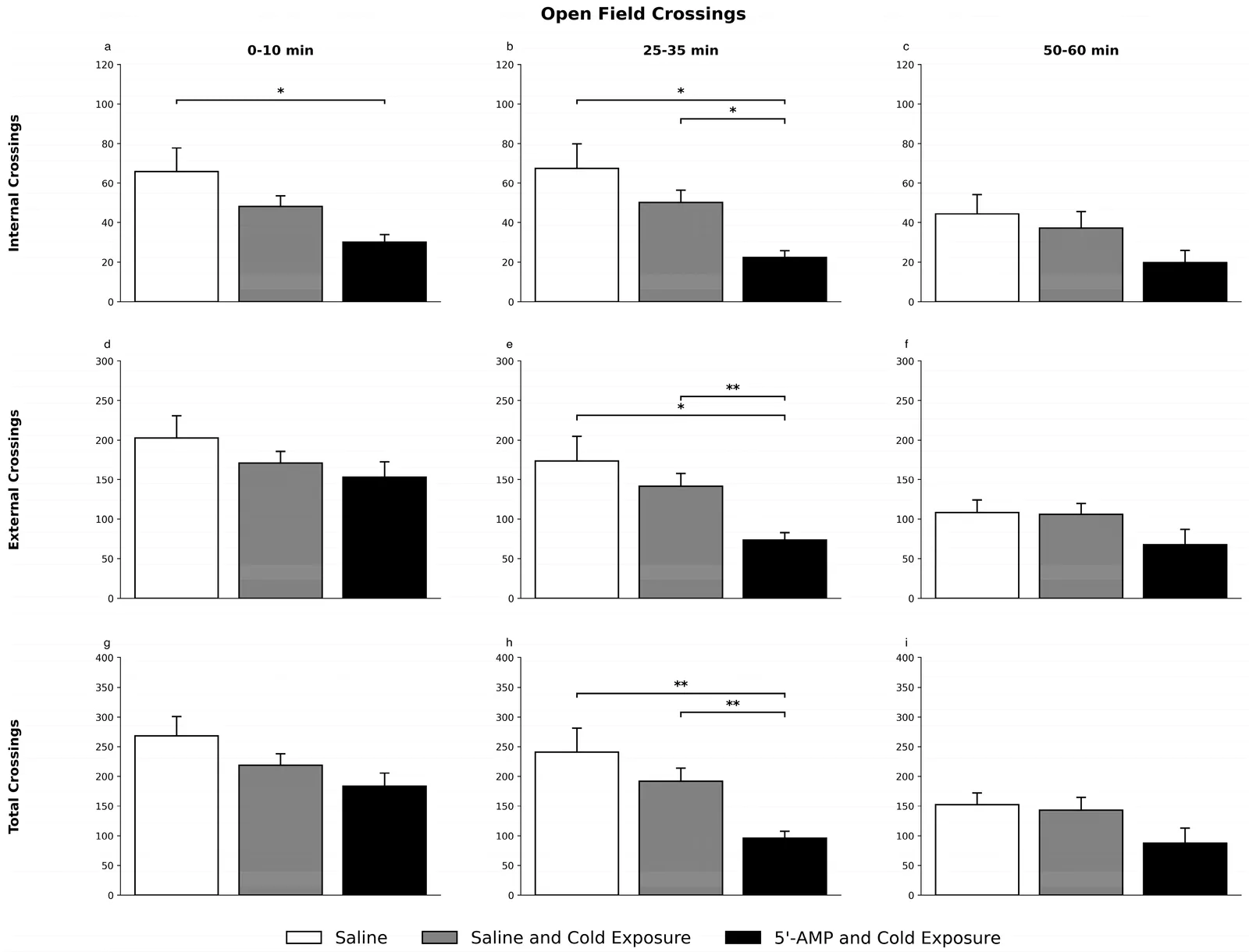
Locomotor activity and spatial distribution of crossings in the open field test after induction of the torpor-like hypothermic state. Internal crossings (**a**–**c**), external crossings (**d**–**f**), and total crossings (**g**–**i**) are shown for three periods of the 60 min session: initial (0 to 10 min; (**a**,**d**,**g**)), central (25 to 35 min; (**b**,**e**,**h**)), and final (50 to 60 min; (**c**,**f**,**i**)). Female C57BL/6 mice were tested upon return to normothermia: Saline at room temperature (*n* = 5, white bars), Saline and Cold Exposure at 15 to 18 °C (*n* = 5, gray bars), or 5′-AMP and Cold Exposure at 15 to 18 °C (*n* = 10, black bars). Bars represent mean ± SEM. One-way ANOVA with Tukey’s HSD post hoc test or Kruskal–Wallis test with Bonferroni-corrected Mann–Whitney U tests were applied as appropriate. * *p* < 0.05, ** *p* < 0.01.

Data on locomotor activity across groups are presented in Figure 2 and Table 2. Behavioral testing began only after each animal had returned to normothermia (cutaneous temperature >=28 °C). Throughout the results, time 0 denotes the onset of the 60 min open field recording, not the time of 5′-AMP injection. 5′-AMP and Cold Exposure animals displayed a progressive decline in locomotor activity compared to both control cohorts. During the initial period (0–10 min), total crossings (grid-line crossings; see Methods) were numerically lower in the 5′-AMP and Cold Exposure cohort but did not reach statistical threshold (*p* = 0.084). By the central period (25–35 min), the reduction had become clear (*p* < 0.001): 5′-AMP and Cold Exposure animals performed 60% fewer total crossings than Saline controls. This suppression persisted into the final period (50–60 min; *p* = 0.036), although pairwise comparisons did not survive Bonferroni correction. These data point to a progressive decline in locomotor activity after the torpor-like hypothermic state, consistent with possible post-arousal fatigue, although fatigue was not directly measured. Alternative explanations are considered in the Section 3.

### 2.3. Spatial Distribution of Activity

Analysis of external (peripheral) versus internal (center) crossings (Figure 2; Table 2) indicated early and sustained reductions in center entries for 5′-AMP and Cold Exposure mice. Internal crossings were already halved during the initial period (*p* = 0.004). This pattern intensified during the central period (*p* < 0.001), when both internal and external crossings were lower for 5′-AMP and Cold Exposure mice than in either control cohort.

Center entries were consistently reduced while peripheral movement was maintained, a pattern indicating heightened thigmotaxis in the 5′-AMP and Cold Exposure animals. Expressed as a fraction of each animal’s total crossings, center occupancy during the initial period was lower for 5′-AMP and Cold Exposure mice (16.6 ± 1.6%) than in Saline controls (24.6 ± 3.1%; F(2,17) = 4.40, η^2^ = 0.34, *p* = 0.029; Tukey *p* = 0.032), indicating a redistribution of movement rather than a proportional reduction across the arena; no group difference in this proportion was present during the central (*p* = 0.155) or final (*p* = 0.407) periods. Mice exposed to cold without 5′-AMP (Saline and Cold Exposure) had numerically intermediate values across multiple measures. No Saline versus Saline and Cold Exposure comparison reached statistical significance, so this ordering is reported as a descriptive pattern consistent with a smaller contribution of cold exposure alone, and not as a demonstrated effect. These results indicate that a short torpor-like hypothermic state diminishes exploratory behavior and redirects movement toward the periphery of the arena.

### 2.4. Ethogram Analysis

**Figure 3:**
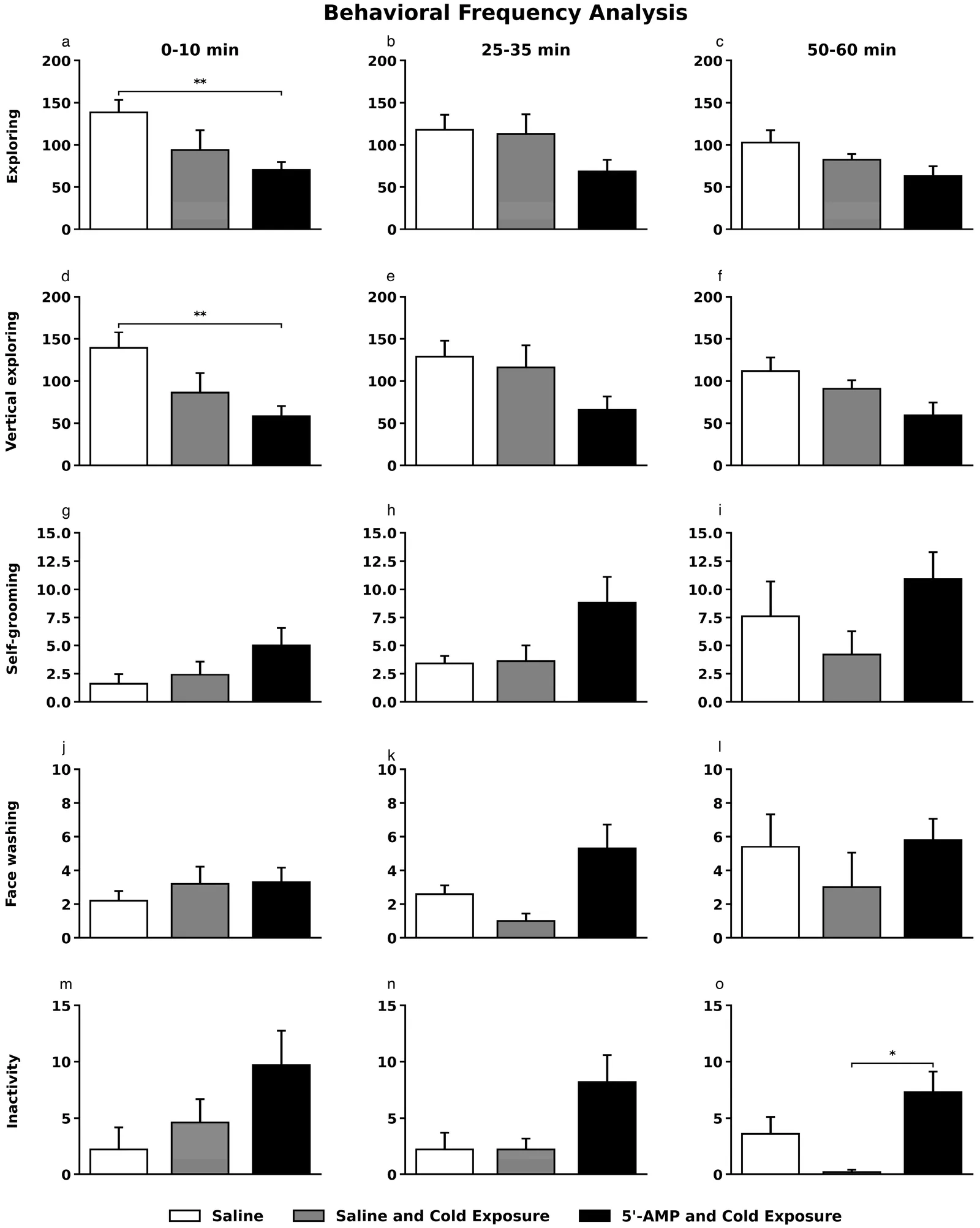
Behavioral bout frequency during the open field test after induction of the torpor-like hypothermic state. Five categories are plotted for the initial, central, and final periods: exploring (**a**–**c**), vertical exploring (**d**–**f**), self-grooming (**g**–**i**), face washing (**j**–**l**), and inactivity (**m**–**o**). Groups and statistical methods as in Figure 2. Bars represent mean ± SEM. * *p* < 0.05, ** *p* < 0.01. Exploratory behaviors fell in the 5′-AMP and Cold Exposure cohort during the initial period (**a**,**d**), while inactivity rose during the final period (**o**).

**Figure 4:**
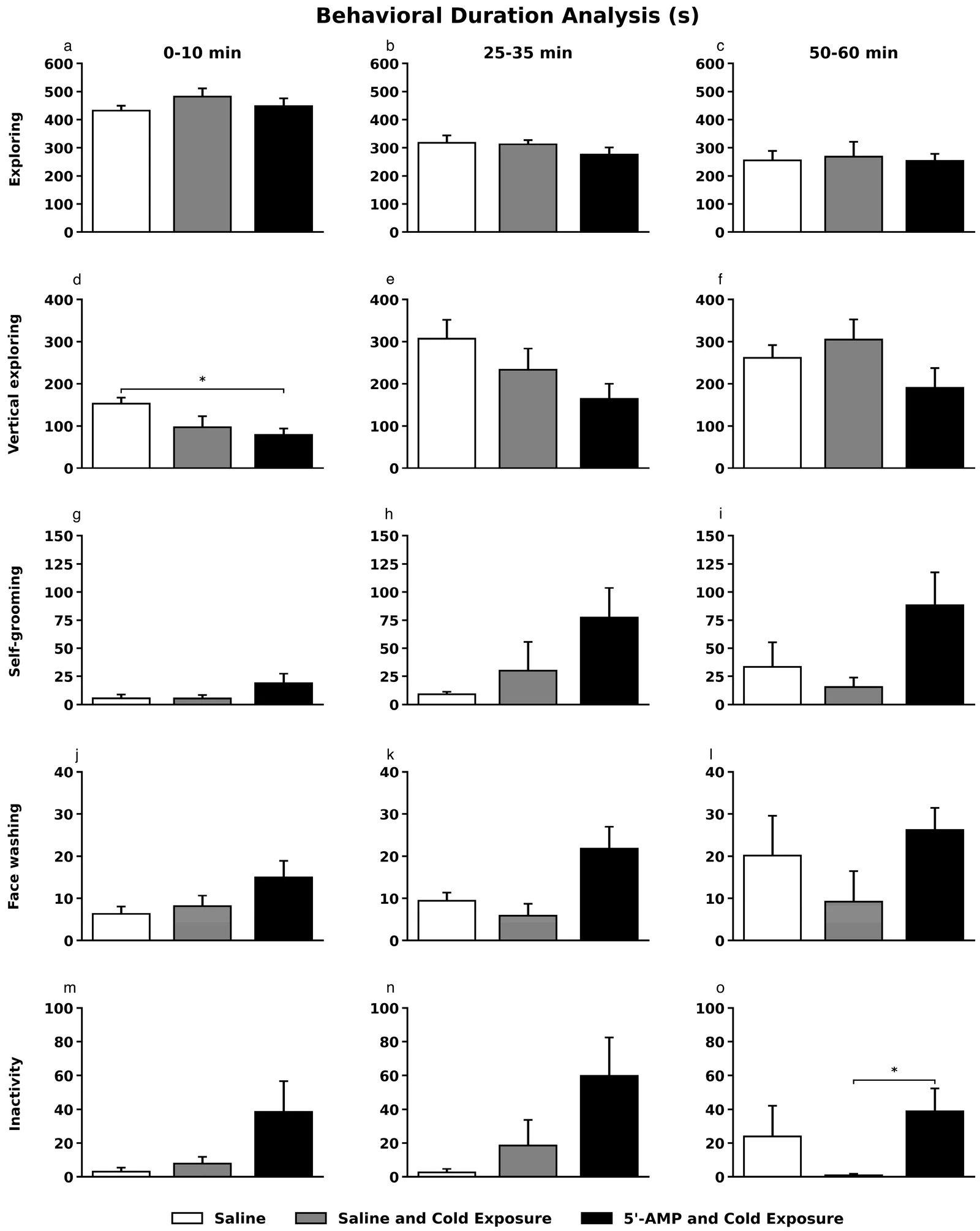
Cumulative behavioral duration during the open field test after induction of the torpor-like hypothermic state. Time (seconds) spent in five categories across the initial, central, and final periods: exploring (**a**–**c**), vertical exploring (**d**–**f**), self-grooming (**g**–**i**), face washing (**j**–**l**), and inactivity (**m**–**o**). Groups and statistical methods as in Figure 2. Bars represent mean ± SEM. * *p* < 0.05. Despite reduced bout frequency, the cumulative time spent exploring remained comparable across groups (**a**–**c**). Because 5′-AMP and Cold Exposure mice reached this comparable total with fewer bouts, their individual exploratory bouts were on average longer.

Over the 60 min open field test, marked behavioral alterations emerged among 5′-AMP and Cold Exposure mice, particularly in vertical exploratory activity and self-maintenance behaviors (Figure 3 and Figure 4; Table 3).

### 2.5. Vertical Exploratory Behaviors (Rearing and Wall Rearing)

Vertical exploring frequency was reduced for 5′-AMP and Cold Exposure mice across the entire session (*p* = 0.029). The largest effect appeared during the initial period (0–10 min; *p* = 0.010), when 5′-AMP and Cold Exposure mice performed fewer than half as many rearing bouts as Saline controls. Vertical exploring duration exhibited a reduction that reached the statistical threshold only during the initial period (*p* = 0.038; Table 3).

### 2.6. General Exploratory Activity

General exploring frequency mirrored the vertical behavior pattern: the 5′-AMP and Cold Exposure cohort initiated fewer exploratory bouts across the full session (*p* = 0.035) and during the initial period (*p* = 0.012). By contrast, the total time spent exploring, that is the cumulative duration of all exploratory bouts summed across the session (Figure 4), did not differ among groups (*p* = 0.707; Table 3). Because 5′-AMP and Cold Exposure mice reached this comparable total in fewer bouts, the mean duration of each individual bout (calculated per animal) was correspondingly longer than in controls (Saline 2.9 ± 0.3 s, Saline and Cold Exposure 4.7 ± 1.6 s, 5′-AMP and Cold Exposure 5.9 ± 1.0 s; Kruskal–Wallis *p* = 0.070). 5′-AMP and Cold Exposure mice therefore explored in fewer but longer episodes rather than for less total time.

### 2.7. Self-Maintenance Behaviors

Self-grooming frequency and duration displayed numerical increases for the 5′-AMP and Cold Exposure cohort across the full session, although these differences did not meet the statistical threshold (frequency: *p* = 0.122; duration: *p* = 0.118; Table 3). During the final period (50–60 min), self-grooming duration was elevated for 5′-AMP and Cold Exposure animals relative to Saline and Cold Exposure controls (*p* = 0.034), though the post hoc comparison did not survive Bonferroni correction (*p* = 0.058). Face washing frequency and duration were numerically higher among 5′-AMP and Cold Exposure mice but did not reach threshold (frequency: *p* = 0.267; duration: *p* = 0.092).

### 2.8. Inactivity

Both inactivity frequency and duration were numerically elevated for the 5′-AMP and Cold Exposure cohort across the entire session (Table 3). However, the full-session omnibus test for inactivity frequency did not reach statistical significance (*p* = 0.055), and no pairwise comparison was significant after post-hoc correction. During the final period (50–60 min), both inactivity frequency (*p* = 0.020) and duration (*p* = 0.041) were elevated. In absolute terms, 5′-AMP and Cold Exposure mice recorded 7.30 ± 1.80 inactive bouts totaling 38.76 ± 13.59 s in this period, compared with 3.60 ± 1.50 bouts totaling 23.86 ± 18.25 s among Saline controls and 0.20 ± 0.20 bouts totaling 0.86 ± 0.86 s among Saline and Cold Exposure controls (Table 3). Because inactivity exhibited a floor effect in the Saline and Cold Exposure group, where only one of five animals contributed the recoded bout, ratios to this control are unstable and are not reported.

### 2.9. Jumping Behavior

Jumping frequency and duration were minimal across all groups and time periods, with no treatment effects detected (*p* = 0.558).

### 2.10. Treatment Group Comparisons

Cold-exposed animals without 5′-AMP (Saline and Cold Exposure) consistently occupied numerically intermediate behavioral values between the Saline and the 5′-AMP and Cold Exposure groups across multiple measures, including vertical exploring frequency, general exploring frequency, and crossings. This graded ordering is consistent with a contribution of cold exposure alone that is smaller and less consistent than that of the full torpor-like hypothermic state. Because no Saline versus Saline and Cold Exposure comparison reached significance, and because the design was not powered to detect effects of this size (see Limitations), the ordering is presented as a hypothesis for adequately powered studies rather than as an established effect.

### 2.11. Y Maze Spontaneous Alternation

**Figure 5:**
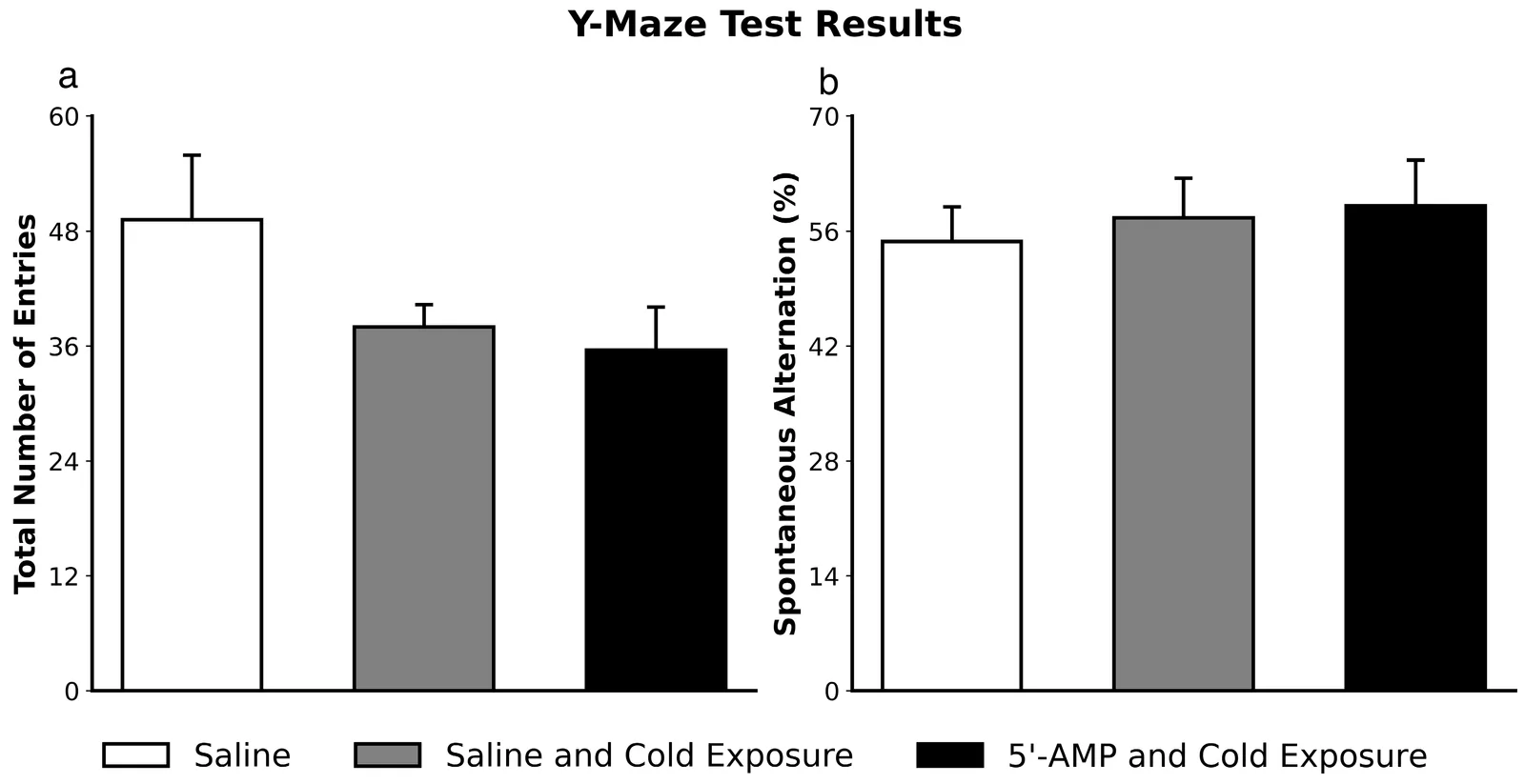
Y-maze performance shows no detectable change in spatial working memory after induction of the torpor-like hypothermic state. (**a**) Total arm entries during the 6 min session. (**b**) Spontaneous alternation percentage. Groups as in Figure 2. Bars represent mean ± SEM. One-way ANOVA detected no between-group differences for either total entries (F(2,17) = 1.93, *p* = 0.176) or spontaneous alternation (F(2,17) = 0.15, *p* = 0.863). All groups performed above the 50% chance level, indicating that the task was performed as expected in every group.

Spatial working memory was assessed using the Y-maze, administered 20 min after the open field session once animals had spontaneously returned to baseline normothermia (Figure 5; Table 4). The total number of arm entries did not differ between groups (*p* = 0.176). Spontaneous alternation likewise did not differ (*p* = 0.863). The 5′-AMP and Cold Exposure cohort achieved 59.10 ± 5.54% alternation, comparable to Saline (54.75 ± 4.23%) and Saline and Cold Exposure (57.62 ± 4.85%) controls. All groups, including the 5′-AMP and Cold Exposure cohort, performed above the 50% chance level. Despite the marked locomotor and exploratory alterations observed in the open field test, the short-duration torpor-like hypothermic state produced no detectable change in this single measure of spatial working memory.

### 2.12. Latency to First Behavior Within the Open Field Session

**Figure 6:**
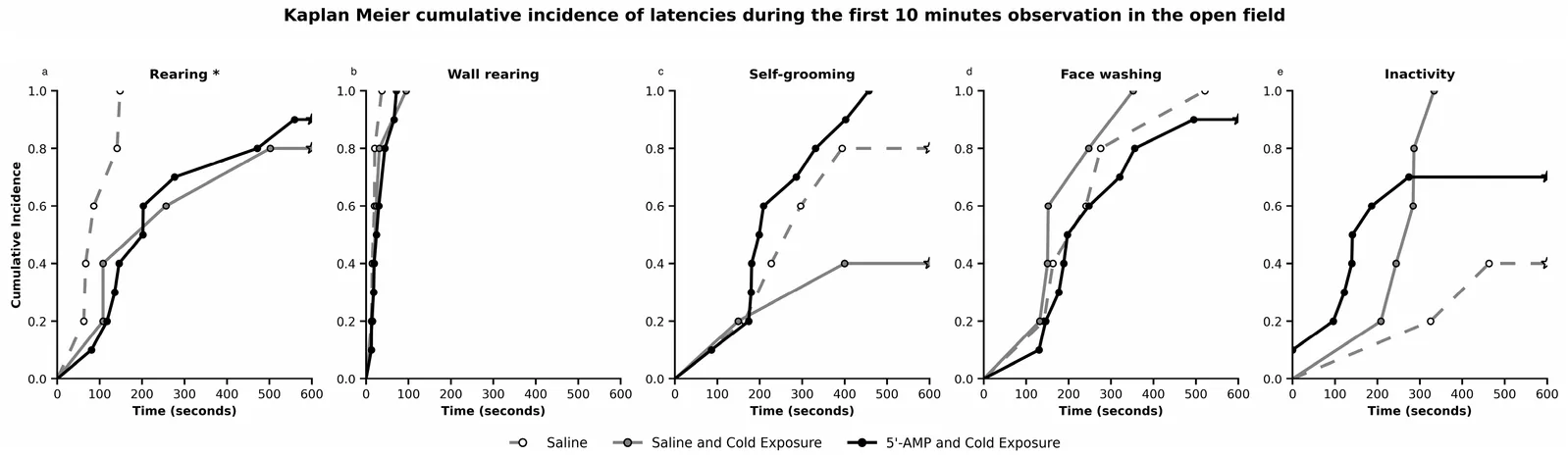
Cumulative incidence curves for behavioral latencies during the initial 10 min open field observation. Time-to-event plots are shown for rearing (**a**), wall rearing (**b**), self-grooming (**c**), face washing (**d**), and inactivity (**e**). Saline (*n* = 5, open circles, dashed line), Saline and Cold Exposure (*n* = 5, gray circles, solid gray line), 5′-AMP and Cold Exposure (*n* = 10, black circles, solid line). Tick marks denote censored observations (behavior not exhibited within 600 s). Log-rank tests with pairwise comparisons were used. * *p* < 0.05 for the overall group effect. This survival-based approach detected treatment effects on behavioral initiation that classical parametric tests missed.

Latency to first occurrence of each behavior, scored from the initial 10 min of the same open field recording and therefore from the same post-arousal, normothermic session rather than a separate test, was analyzed using survival methods, as recommended by Jahn-Eimermacher et al. [43]. Survival analysis properly handles censored observations and provides interpretable effect sizes through hazard ratios (Figure 6; Table 5).

Rearing latency differed among groups (log-rank *p* = 0.022). Both the 5′-AMP and Cold Exposure and the Saline and Cold Exposure mice displayed substantially delayed onset of vertical exploratory behavior compared to Saline controls, with 77% and 82% reductions in the rate of rearing initiation, respectively (Table 5). Saline animals initiated rearing at a median of 86.0 s, compared to 201.9 s (5′-AMP and Cold Exposure) and 256.1 s (Saline and Cold Exposure). Pairwise comparison confirmed a difference between the Saline and the 5′-AMP and Cold Exposure animals (*p* = 0.014).

Self-grooming latency did not differ significantly among groups (log-rank *p* = 0.076), although a nominal pairwise difference was present between the 5′-AMP and Cold Exposure and the Saline and Cold Exposure animals (*p* = 0.029). All 5′-AMP and Cold Exposure mice initiated grooming (10/10, median = 198.6 s), compared to partial penetrance in Saline and Cold Exposure (2/5, median not reached) and Saline (4/5, median = 296.4 s) groups.

There was a trend toward accelerated onset of inactivity across treatment conditions (log-rank *p* = 0.146). Only 40% of Saline-treated animals (2/5) became inactive during the 600 s observation period, whereas all Saline and Cold Exposure animals (5/5, median = 284.0 s) and 70% of 5′-AMP and Cold Exposure animals (7/10, median = 141.0 s) became inactive. Pairwise analysis confirmed a difference between Saline and Saline and Cold Exposure groups (*p* = 0.006).

No differences were detected in wall rearing latency (*p* = 0.385) or face washing latency (*p* = 0.474). Wall rearing was initiated rapidly across all groups (Saline: 19.6 s; Saline and Cold Exposure: 23.9 s; 5′-AMP and Cold Exposure: 24.4 s), and face washing latencies were comparable across conditions (Table 5). All animals performed wall rearing and face washing within the observation period, which confirms preservation of these behaviors irrespective of treatment.

Survival analysis demonstrated greater statistical power than classical approaches: the log-rank test detected treatment effects on rearing latency (*p* = 0.022) that were not identified by ANOVA (*p* = 0.144), and pairwise comparisons identified differences in self-grooming and inactivity that classical post hoc tests failed to detect. This enhanced sensitivity is attributable to appropriate handling of the substantial censored data fraction, particularly in behaviors with low penetrance, such as inactivity (60% censoring in Saline controls).

## 3. Discussion

Intraperitoneal administration of 5′-AMP (400 mg/kg) combined with a reduced ambient temperature (15–18 °C) reliably induced the reversible torpor-like hypothermic state in female C57BL/6 mice. All treated animals entered deep hypothermia, reaching a mean cutaneous temperature nadir of 18.92 ± 0.34 °C (individual minima 17.80 to 21.30 °C; CV = 5.4%) and spontaneously rewarmed to normothermia within approximately 400 min (range 190 to 480 min), with no external heating intervention required. These temperature dynamics are similar to those reported for adenosine-mediated torpor models [9,10] and for synthetic torpor induced by rostral ventromedial medulla (RVMM) inhibition in rats [7]. Of the four cardinal features of torpor, namely reduced body temperature, heart rate, respiratory rate, and energy expenditure [1,2,3], only a cutaneous proxy for the first was recorded here, so the correspondence we can claim with those models is thermal rather than physiological. We did not measure oxygen consumption in this cohort. As outlined in the Introduction, the hypometabolic character of the 5′-AMP state rests on prior indirect calorimetry in the same model [23,24,25,44], and whether the metabolic decline is fully regulated or partly a passive consequence of cooling remains debated [27,28,29]. Because we recorded cutaneous temperature rather than metabolic rate, our data do not by themselves resolve this question, and concurrent calorimetry is the priority for future work.

### 3.1. Locomotor and Exploratory Behavior

Upon return to normothermia, 5′-AMP and Cold Exposure mice displayed a progressive decline in locomotor activity across the 60 min open field session. Although the initial period (0–10 min) showed no trend toward reduced crossings, the central period (25–35 min) revealed a marked reduction that reached statistical significance (*p* < 0.001; Table 2), a pattern that persisted into the final period. This temporal profile is consistent with possible post-arousal fatigue, although fatigue was not directly measured. The energy cost of shivering thermogenesis during rewarming places a metabolic burden on skeletal muscle [17,18] and could have depleted muscular energy reserves before testing began. However, heightened anxiety-like behavior, residual hypothermia, and a transient sedative action of 5′-AMP are equally compatible with these data.

Two features of the data argue against a cognitive deficit model, whichever of these mechanisms proves correct. First, although 5′-AMP and Cold Exposure mice initiated fewer exploratory bouts (*p* = 0.035; Table 3), the total time they spent exploring was unchanged (*p* = 0.707; Table 3). Each bout was therefore longer on average, the arithmetic consequence of a preserved total distributed over fewer bouts.

Together with the concurrent reduction of roughly 60% in locomotor crossings (*p* < 0.001; Table 2), this indicates that exploratory drive remained intact while motor output was reduced and slowed. Because the exploring category combines locomotion and stationary investigation such as sniffing, the longer bouts are consistent with slower movement, a shift toward more in-place investigation, or both. In each case, exploratory motivation is preserved while motor output is diminished. Second, survival analysis of behavioral latencies showed that wall rearing, the first exploratory behavior to appear, was initiated equally rapidly across all groups (median: 19.6–24.4 s). This confirms intact motivation to explore the novel environment.

### 3.2. Spatial Distribution and Thigmotaxis

A consistent reduction in center entries, combined with maintained peripheral movement, indicates heightened thigmotaxis in 5′-AMP and Cold Exposure animals. We interpret this primarily as anxiety-like behavior. Thigmotaxis in mice is increased by anxiogenic drugs and reduced by anxiolytic drugs [45]. Here, the early effect was a redistribution of movement toward the walls rather than a proportional loss of activity, which a purely energetic account would not predict. The open field test alone, however, cannot establish an anxious state. Thigmotaxis is a nonspecific measure, and these data are consistent with both possible anxiety-like behavior and energy conservation strategies. By restricting movement to the periphery, animals also minimized the total distance traveled while maintaining surveillance of the environment. The later decline, which was proportionally uniform, is more consistent with the general post-arousal reduction in activity discussed above. Confirming an anxiety-like state would require a dedicated paradigm, such as the elevated plus maze or the light/dark box.

### 3.3. Self-Maintenance Behaviors and Thermoregulatory Coping

Self-grooming frequency and duration showed numerical increases in the 5′-AMP and Cold Exposure group across the session and reached the detection threshold for duration during the final period (*p* = 0.034; Table 3). Rodent grooming serves both thermoregulatory and stress-displacement functions. The concurrent increase in grooming and face-washing, together with complete penetrance of grooming in the 5′-AMP and Cold Exposure group (10/10 animals), is consistent with post-arousal mice engaging in thermoregulatory restoration. However, because grooming also serves as stress displacement and body temperature was not recorded during the open field session, we cannot distinguish the two. The co-occurrence of reduced locomotion, heightened thigmotaxis, and increased thermoregulatory grooming in these animals resembles the combined thermoregulatory and anxiety-like profile shaped by GABAergic circuits in pharmacological models. In these models, GABA-A receptor signaling dissociates into an alpha-1 component linked to thermoregulation and locomotor sedation and an alpha-2/alpha-3 component linked to anxiolysis [46]. We did not measure any GABAergic readout in this study, so whether residual perturbation of these pathways contributes to the post-arousal phenotype remains an untested hypothesis that targeted receptor or electrophysiological work could address in future studies.

### 3.4. Spatial Working Memory

Despite the marked locomotor and exploratory changes, spontaneous alternation was unaffected. It was statistically indistinguishable across groups (*p* = 0.863; Table 4), with all groups performing above the 50% chance level. The absence of a detectable effect on this hippocampal-dependent task after a state known to induce reversible tau hyperphosphorylation [20] is reassuring, but spontaneous alternation probes only one aspect of hippocampal function, and no other cognitive domain was assessed. Because abnormal tau hyperphosphorylation is a defining feature of Alzheimer’s disease, its appearance during natural and synthetic torpor has raised concerns about potential neuronal damage [20]. In torpid mice, however, both the hyperphosphorylation and the associated somato-dendritic tau accumulation reverse completely upon arousal, including in animals expressing human tau [21]. Furthermore, the phospho-sites involved are not accompanied by the tangle-associated microtubule-binding-region fragments seen in Alzheimer’s disease [22]. Our behavioral findings are consistent with that picture, although tau was not measured in this cohort and no causal link can be drawn. A single, short-duration episode of the torpor-like hypothermic state left spontaneous alternation unaffected. Whether repeated or longer episodes, or pharmacological induction specifically, preserve this profile remains to be established.

Under the conditions tested, the Y-maze results show no detectable impairment of spatial working memory through the torpor-like hypothermic state and subsequent arousal. However, the present sample size limits the detection of small effects, and spontaneous alternation indexes only one hippocampal-dependent process.

### 3.5. Cold Exposure as an Independent Variable

The inclusion of a Saline and Cold Exposure control group enabled us to distinguish effects specific to the torpor-like hypothermic state from those that are attributable to cold stress alone. Across multiple measures (crossings, vertical exploring frequency, behavioral latencies), the Saline and Cold Exposure group consistently exhibited intermediate values between Saline controls and the 5′-AMP and Cold Exposure group. This graded response confirms that ambient temperature reduction alone exerts measurable behavioral effects, though these are less pronounced and less consistent than those that are produced by pharmacological torpor induction.

Survival analysis of behavioral latencies revealed that cold exposure alone accelerated the onset of inactivity compared to room-temperature controls (*p* = 0.006; Table 5), even without 5′-AMP administration. This finding confirms that low ambient temperature during the observation period is itself a behavioral stressor that must be controlled for in torpor studies.

### 3.6. Translational Relevance

These findings contribute to the safety assessment of the 5′-AMP-induced torpor-like hypothermic state for translational applications, particularly in spaceflight and critical care medicine. Radiation is one of several hazards of long-duration missions for which controlled hypometabolism has been proposed as a countermeasure [17,37,40], and the state induced by 5′-AMP has been shown to confer radioprotection against accelerated carbon ions in mice [39]. The present study adds behavioral evidence that a single, short-duration episode of the torpor-like hypothermic state does not detectably impair the neurocognitive functions assessed here, despite the known cellular-level brain modifications, and begins to fill a gap in the translational pathway. The scope of this evidence is nonetheless narrow: one induction episode, twenty female mice, one cognitive task, and no direct measurement of metabolic rate. It constrains the size of any acute neurobehavioral deficit rather than establishing translational safety.

Beyond spaceflight, the 5′-AMP-induced torpor-like hypothermic state holds promise for extending therapeutic intervention windows in time-sensitive clinical emergencies, such as stroke, cardiac arrest, and trauma, where metabolic suppression could limit ischemic injury [18]. The differential metabolic response between normal and neoplastic tissues opens a further avenue for radioprotection during cancer therapy [41].

## 4. Limitations

Several limitations apply. Only female mice were used, so sex-dependent variability in torpor susceptibility, reported previously for fasting-induced torpor, could not be assessed [15]. The small sample sizes (*n* = 5 per control group, *n* = 10 for 5′-AMP and Cold Exposure) limited statistical power: at alpha = 0.05 this design has 80% power only for very large effects (η^2^ >= 0.37) and about 30% power for a conventionally large effect (η^2^ = 0.14). Non-significant results are therefore inconclusive rather than evidence of no effect and are not interpreted biologically; effect sizes for every omnibus test are reported in Table 2, Table 3 and Table 4. Several control-group means in the final period were near zero, so ratios there are unstable and the absolute values in Table 3 are preferred. Invasive sampling was precluded by the need for mice to complete the behavioral battery, so biochemical markers such as site-specific tau phosphorylation could not be correlated with behavior. Without histological and molecular endpoints, these data cannot speak to tau pathology, synaptic integrity, or neuroinflammation. Only a single episode was induced, so the effects of repeated inductions are unknown. Cognitive assessment was limited to spontaneous alternation, a single index of spatial working memory; other hippocampal-dependent and non-hippocampal domains were not tested, so these data cannot speak to cognition beyond this task. Temperature was recorded using cutaneous infrared thermometry rather than implanted telemetry, a noninvasive choice that is less precise as an estimate of core temperature [47]. Skin and core temperature diverge whenever peripheral vasoconstriction is engaged, as in cold exposure and hypometabolic states, so cutaneous temperature cannot index the depth of a torpor-like state. The present values establish that a deep, reversible hypothermic episode occurred, but not how far core temperature fell. The direction of that discrepancy is conservative, since subcutaneous and core temperatures converge as body temperature falls in mice undergoing daily torpor [48], although too variably to convert surface into core values. Direct core-temperature telemetry with simultaneous indirect calorimetry is required to quantify the depth and metabolic character of this state. Cutaneous temperature in cold-exposed controls was logged in a three-animal subset. Because all animals were fed ad libitum, under which C57BL/6 mice remain normothermic, this documents control thermal stability. Metabolic rate was not measured; the hypometabolic character of the state is inferred from prior indirect calorimetry in this model [23,24]. Because a sub-thermoneutral ambient temperature and temperature-only monitoring can both overstate apparent metabolic suppression [29], we cannot yet distinguish a regulated, actively defended hypometabolic state from a pharmacologically forced hypothermic response. More broadly, this was a behavioral study by design; heart rate, respiratory rate, oxygen consumption, plasma corticosterone, and markers of neuronal injury, neuroinflammation, and synaptic integrity were all unmeasured. Their absence does not affect the validity of the behavioral findings but limits what can be concluded about the state itself. The increases in thigmotaxis and self-directed behavior are accordingly described in the sense conventional for the open field test rather than as a demonstrated stress response. A companion cohort instrumented for telemetry, indirect calorimetry, serial corticosterone, and histology is the necessary next step. Finally, because rewarming imposes a metabolic cost on skeletal muscle, a transient effect of energy state on locomotor performance cannot be excluded, although food and water were withheld identically across groups and testing began only after normothermia.

Behavioral testing also fell within the animals’ dark (active) phase, under dim red light, because the colony was maintained on a reversed light/dark cycle. This strengthens the interpretation since reduced activity was measured against an active-phase baseline, controls were tested at matched clock times, and all testing preceded the active-to-rest transition.

## 5. Conclusions

Pharmacological induction of a torpor-like hypothermic state with 5′-AMP produced consistent, reversible hypothermia in female C57BL/6 mice. Behavioral changes accompanied the arousal phase, but the intervention did not produce detectable impairments in this short-term behavioral battery. Locomotor activity and vertical exploration were reduced during the post-arousal period, consistent with possible post-arousal fatigue, although the mechanism was not directly measured. No impairment of spatial working memory was detected using the Y-maze. Increased thigmotaxis during the early post-arousal period is most consistent with an anxiety-like response, although the open field test alone cannot establish an anxious state, and self-grooming showed a numerical, non-significant increase. However, these effects were modest and did not disrupt fundamental behavioral organization. Neurocognitive safety cannot be established from a single behavioral task after a single exposure, and the present design included no histological or molecular assessment.

These results are a first behavioral step in evaluating the neurobehavioral safety of a short-duration, single-episode torpor-like hypothermic state and motivate, but do not by themselves justify, translational development. For long-duration spaceflight, controlled metabolic suppression could reduce crew resource requirements (food, water, and oxygen), attenuate muscle and bone loss from microgravity, confer radioprotection against simulated space radiation, including accelerated heavy ions [37,38,39,41], and mitigate the psychological burden of prolonged confinement [40]. In clinical medicine, the ability to reversibly suppress metabolism opens therapeutic avenues for extending intervention windows in stroke, cardiac arrest, and trauma, where even modest reductions in metabolic demand preserve organ viability.

Future work should address the effects of repeated inductions of the torpor-like hypothermic state, include both sexes, correlate behavioral outcomes with molecular markers of neural integrity (including tau phosphorylation and aggregation dynamics [22]), and systematically vary its depth and duration. If these investigations confirm and extend the absence of detectable impairment reported here, the 5′-AMP-induced torpor-like hypothermic state may become a viable biomedical tool for both terrestrial medicine and human space exploration.

## 6. Materials and Methods

This study was designed and reported in accordance with the ARRIVE guidelines [49]. Animals were randomly allocated to experimental groups. All procedures were approved by the Institutional Animal Care and Use Committee and conducted in compliance with European and national legislation. Sample sizes were defined prospectively on the basis of previous experience with the 5′-AMP model and the exploratory nature of the study. Because the 5′-AMP-induced state is characterized by substantial inter-individual variability in its induction and recovery kinetics, the treated group was deliberately sampled at twice the size of each control group (*n* = 10 vs. *n* = 5) to obtain a more representative estimate of the behavioral response after 5′-AMP exposure. Control-group sizes were kept at the minimum considered sufficient to provide reference values for vehicle treatment and cold exposure, in accordance with the 3Rs principle. No single primary outcome was pre-specified for an a priori power calculation. All animals meeting the inclusion criteria were included in the analyses, and no data were excluded. Behavioral assessments were conducted from direct observation and video recordings using standardized ethological scoring methods. Key experimental conditions, including housing, treatment, experimental protocol and outcome measures, are reported to ensure transparency and reproducibility.

### 6.1. Animal Models and Experimental Groups

Twenty female C57BL/6 mice were purchased from ENVIGO RMS SRL (Udine, Italy). Females were used for consistency with the study in which 5′-AMP-induced hypometabolism was established and dose characterized, which was performed in female C57BL/6 mice [23]. Restricting the cohort to a single sex also reduced variability between animals in this exploratory pilot; the effect of sex on these outcomes remains to be determined (see Section 4). Upon arrival (28 to 30 days old, nulliparous), animals were housed in a temperature-controlled room at 21 ± 1 degrees C (60 ± 10% relative humidity) on a reversed 12:12-h light/dark cycle. White lights were on from 19:30 to 07:30 (the animals’ light/rest phase), while from 07:30 to 19:30 the room was illuminated only with dim red light, which mice cannot perceive and therefore constitutes their dark (active) phase. All husbandry and behavioral testing were performed during this red-lit active phase. Mice were group-housed (three per cage) in plastic cages (42 × 27 × 14 cm) with metal tops and floor bedding. Ad libitum access to standard rodent chow (Mucedola, Settimo Milanese, Italy) and water was provided throughout the study. Bedding and enrichment were replaced weekly. All procedures followed European Community guidelines (Directive 2010/63/EU) and Italian legislation (D.Lgs. 26/2014).

Mice were randomly assigned to three experimental groups: (1) 5′-AMP and Cold Exposure (*n* = 10), in which 5′-AMP (400 mg/kg) was injected intraperitoneally and animals were housed at 15 to 18 degrees C until spontaneous return to normothermia; (2) Saline and Cold Exposure (*n* = 5), in which an equivalent volume of vehicle was injected intraperitoneally and animals were housed at 15 to 18 degrees C; and (3) Saline (*n* = 5), in which an equivalent volume of vehicle was injected intraperitoneally and animals were housed at 22 degrees C.

### 6.2. Drug Preparation

5′-AMP (Adenosine 5′-monophosphate sodium salt; Sigma Aldrich, St. Louis, MO, USA) was provided as a powder and dissolved in double-distilled water to a concentration of 25 mg/mL. The resulting acidic solution was then titrated with 1 M NaOH to a final pH of 7.0, which was expected to guarantee physiological compatibility prior to injection.

### 6.3. Induction and Monitoring of the Torpor-like Hypothermic State

The torpor-like hypothermic state was induced by intraperitoneal injection of 5′-AMP at 400 mg/kg. The solution was prepared fresh, brought to room temperature, and adjusted according to each mouse’s body weight measured immediately before injection. Mice in the 5′-AMP and Cold Exposure group received 5′-AMP and were then placed in cages maintained at 15 to 18 degrees C. Mice in the Saline and Cold Exposure group received an equivalent volume of vehicle (mL/kg) and were placed at 15 to 18 degrees C. Saline controls received vehicle only and remained at 22 degrees C.

During the torpor-like hypothermic state, dorsal interscapular skin temperature was recorded every 10 min using a non-contact infrared thermometer (ERICKHILL 600C). Skin temperature served as the sole in-study physiological index of the torpor-like state; no cardiovascular, respiratory, endocrine, or molecular variable was recorded. Furthermore, metabolic rate (oxygen consumption) was not measured in this cohort; the hypometabolic character of the state is inferred from cutaneous temperature together with prior indirect-calorimetry studies in the same model (see Limitations). Throughout this manuscript, “torpor-like hypothermic state” denotes the state induced by 5′-AMP at a reduced ambient temperature, defined operationally here by cutaneous temperature alone. This term does not assert metabolic equivalence with natural torpor.

### 6.4. Experimental Timeline

The experimental protocol followed a structured timeline to help maintain consistent conditions across all behavioral assessments. On Day 0, injections were administered at 10:00. Mice received either 5′-AMP (400 mg/kg, i.p.) or vehicle and were immediately placed in temperature-controlled conditions (15 to 18 degrees C for the 5′-AMP and Cold Exposure and the Saline and Cold Exposure groups; 22 degrees C for Saline controls). Food and water were available ad libitum until the time of injection, after which they were withheld during cold exposure, arousal, and the subsequent behavioral testing. This procedure was identical for all animals and all groups. Standard chow and water were returned to the home cage immediately after testing concluded, and behavioral testing was initiated only after animals had regained normothermia. Behavioral testing began only after each animal had spontaneously rewarmed to its own pre-treatment baseline temperature. No external heating was applied and temperature was not statistically normalized or used as a covariate. Immediately upon this spontaneous return to normothermia (cutaneous temperature at least 28 degrees C, consistent with the pre-injection baseline of 29.13 ± 0.24 degrees C; mean time to return 397.0 ± 26.8 min post-injection), each mouse was transferred to the open field arena for 60 min of behavioral observation. The Y-maze followed in immediate succession, 20 min after open field completion and within the same normothermic recovery window, so surface temperature had returned to baseline before both assessments. Control groups followed an identical timeline. Behavioral testing was initiated at time-matched intervals that corresponded to the 5′-AMP and Cold Exposure group’s recovery period. Because all injections were administered at 10:00 and the interval to spontaneous normothermia varied between individuals (190 to 480 min), open field testing began between approximately 13:10 and 18:00. Owing to the reversed light/dark cycle (see Section 6.1), this entire window fell within the animals’ dark (active) phase, the period of peak spontaneous locomotor activity in nocturnal rodents, and testing was conducted under dim red illumination. Even the latest-finishing animal completed both the open field and Y-maze assessments by approximately 19:26, before the 19:30 dark-to-light (active-to-rest) transition, so no behavioral assessment occurred during the rest phase. All three groups were tested within this same active-phase window, and each control animal was tested at a clock time matched to a 5′-AMP and Cold Exposure animal. Therefore, the circadian phase was balanced across groups by design.

### 6.5. Behavioral Tests

#### Open Field

Upon spontaneous return to its pre-treatment baseline temperature (normothermia, cutaneous temperature at least 28 degrees C), each mouse was placed in a black-walled acrylic arena (30 × 30 × 30 cm) and recorded for 60 min. The start of this recording defines time 0 for all open field and ethogram measures, so the behavioral time axis is referenced to each animal’s return to normothermia rather than to the time of 5′-AMP injection. Because the interval from injection to normothermia varied between individuals (range 190 to 480 min; Figure 1), referencing time 0 to recovered normothermia ensured that every mouse, including the time-matched control groups, was assessed from a common physiological starting point rather than a common time after injection. This test assessed locomotion, exploration, and anxiety-like responses to a novel environment. The arena bore no physical markings. For locomotor quantification, a standardized 16-cell grid, identical in cell dimensions and position for every animal, was overlaid on the video image in BORIS, and crossings of the grid lines were counted and classified as either external (peripheral) or internal (center) to evaluate thigmotaxis. Applying the same fixed grid to every recording ensured that the quadrant size and location were constant across all mice and groups. Video recordings were analyzed using BORIS software (Behavioral Observation Research Interactive Software). Each behavioral category was assigned a keyboard key, which allowed the observer to log frequency and duration. The standardized ethogram (Table 1) included wall rearing (standing on hind legs with forepaws against the wall), rearing (standing upright supported only by hind legs), general exploratory behavior not otherwise classified (e.g., sniffing, locomotion), jumping (leaping within the arena), inactivity (absence of movement for more than three seconds), self-grooming (self-cleaning behavior), and face washing (cleaning of the facial region). For statistical analysis, wall rearing and rearing were combined into a single category termed “vertical exploring” to simplify comparisons. Data analysis focused on three 10 min segments: initial (0 to 10 min), middle (25 to 35 min), and final (50 to 60 min). Each segment was further subdivided into five 2 min bins for temporal comparisons of behavior frequency and duration. Behavioral scoring was performed by an observer blind to group allocation.

In addition to frequency and duration, the latency to first occurrence of each ethogram behavior was scored from the same open field recordings within the initial 10 min segment (time-to-event, censored at 600 s) and analyzed by survival methods (see Statistics and reproducibility). The latency analysis was therefore not a separate behavioral test but an additional readout extracted from the open field session, sharing its identical post-arousal, normothermic timing.

### 6.6. Y-Maze

Twenty minutes after open field testing, with animals still in the post-arousal normothermic state at baseline temperature, mice were placed in a Y-shaped maze constructed of gray acrylic (arm dimensions: 35 × 5 × 10 cm; three identical arms at 120 degree angles from a central platform). Each mouse was placed in the center and allowed to explore freely for six minutes. An arm entry was recorded when all four paws had entered an arm. The number and sequence of arm entries were recorded. Spontaneous alternation was calculated as the percentage of consecutive triads that contained entries into all three arms [number of alternations/(total entries-2) × 100]. This test exploits rodents’ natural tendency to explore novel arms rather than return to recently visited ones, and the resulting ratio provides an index of spatial working memory.

### 6.7. Statistics and Reproducibility

Data are presented as the mean ± SEM. Statistical analyses were performed in Python (version 3.12.2) using SciPy (version 1.16.2), statsmodels (version 0.14.6), and lifelines (version 0.30.0), with pandas (version 2.3.3) for data handling and matplotlib (version 3.10.7)/seaborn (version 0.13.2) for visualization. All tests were two-tailed with alpha = 0.05. For each analysis, the test used, sample size (*n* per group), exact *p* value (*p*) are reported.

Normality was assessed with the Shapiro–Wilk test and homoscedasticity with Levene’s test. When assumptions were met (*p* > 0.05), a one-way ANOVA was applied to compare the three independent groups, followed by Tukey’s honest significant difference (HSD) post hoc test. When assumptions were violated, the non-parametric Kruskal–Wallis H test was used, followed by two-tailed Mann–Whitney U tests with Bonferroni correction (corrected *p* = raw *p* × 3, capped at 1.0). Crossings and spatial distribution data were analyzed by one-way ANOVA or Kruskal–Wallis tests as above; the choice between tests followed the same normality and variance criteria. Effect sizes for the omnibus tests are reported alongside each test statistic in Table 2, Table 3 and Table 4: eta squared (η^2^) for one-way ANOVA, computed as η^2^ = (F × df1)/(F × df1 + df2), and epsilon squared (ε^2^) for the Kruskal–Wallis test, computed as ε^2^ = (H − k + 1)/(*n* − k), where k is the number of groups and *n* the total sample size; negative ε^2^ estimates were truncated to zero. For the latency analyses, hazard ratios with 95% confidence intervals (Table 5) serve the same purpose.

We analyzed ethogram behaviors (frequency and duration) with the same parametric or non-parametric framework across three 10 min windows (0 to 10, 25 to 35, and 50 to 60 min) and across the full 60 min session. For Y-maze performance (total entries and spontaneous alternations), we applied one-way ANOVA after confirming normality and equal variances. We analyzed latency measures with survival-analysis methods [43]. Group differences were assessed by log-rank test, followed by pairwise log-rank comparisons when the omnibus test reached the significance threshold (*p* < 0.05). Cox proportional-hazards regression estimated hazard ratios (HR) relative to Saline controls, where HR > 1 indicates faster behavior initiation and HR < 1 indicates delayed initiation. Plots show full data distributions with individual points or box-and-whisker plots.

## Figures and Tables

**Table 1:** Ethogram of behavioral categories scored during the open field test. Behaviors were logged for frequency and duration with Behavioral Observation Research Interactive Software (BORIS, versio 9.13). Wall rearing and rearing were combined into “vertical exploring” for analysis, as the two appear to reflect a single motivational state. See Section 6 for definitions.

Behavior	Description
Wall rearing	Standing on hind legs with forepaws against the arena wall
Rearing	Standing upright supported only by hind legs, without wall contact
Exploring	General exploratory behavior not otherwise classified (e.g., sniffing, locomotion)
Jumping	Leaping within the arena
Inactivity	Absence of movement for more than three seconds
Self-grooming	Self-cleaning behavior directed at the body
Face washing	Cleaning of the facial region

For statistical analysis, wall rearing and rearing were combined into *“vertical exploring.”*

**Table 2:** Locomotor activity across time blocks in the open field test. Data represent mean ± SEM. Saline (*n* = 5), Saline and Cold Exposure (*n* = 5), 5′-AMP and Cold Exposure (*n* = 10). When normality (Shapiro–Wilk) and homoscedasticity (Levene’s test) assumptions held, a one-way ANOVA with Tukey’s HSD post hoc test was applied; otherwise, the Kruskal–Wallis H test followed by Bonferroni-corrected pairwise Mann–Whitney U tests was used. Three 10 min blocks were analyzed: initial (0 to 10 min), central (25 to 35 min), and final (50 to 60 min). This temporal resolution may capture progressive fatigue effects. Each omnibus test statistic is followed by its effect size (η^2^ for ANOVA, ε^2^ for Kruskal–Wallis test; see Methods). * *p* < 0.05.

**A. Total crossings**						
**Time Block**	**Saline**	**Saline and Cold Exposure**	**5′-AMP and Cold Exposure**	**Test Statistic**	***p***	**Post Hoc**
Initial (0–10 min)	268.60 ± 32.28	219.00 ± 18.97	183.30 ± 22.14	F(2,17) = 2.88, η^2^ = 0.25	0.084	-
Central (25–35 min)	240.80 ± 40.66	191.80 ± 22.23	96.00 ± 11.63	F(2,17) = 12.08, η^2^ = 0.59	<0.001	Saline vs. 5′-AMP and Cold Exposure *p* < 0.001 *; Saline and Cold Exposure vs. 5′-AMP and Cold Exposure *p* = 0.018 *; Saline vs. Saline and Cold Exposure *p* = 0.383
Final (50–60 min)	152.40 ± 19.90	143.00 ± 21.75	87.30 ± 25.50	H(2) = 6.63, ε^2^ = 0.27	0.036	Saline vs. 5′-AMP and Cold Exposure *p* = 0.166; Saline and Cold Exposure vs. 5′-AMP and Cold Exposure *p* = 0.084; Saline vs. Saline and Cold Exposure *p* = 1.000 (Bonferroni)
**B. Internal (center) crossings**						
**Time Block**	**Saline**	**Saline and Cold Exposure**	**5′-AMP and Cold Exposure**	**Test Statistic**	***p***	**Post Hoc**
Initial (0–10 min)	65.80 ± 11.96	48.20 ± 5.36	30.10 ± 3.82	F(2,17) = 7.86, η^2^ = 0.48	0.004	Saline vs. 5′-AMP and Cold Exposure *p* = 0.003 *; Saline and Cold Exposure vs. 5′-AMP and Cold Exposure *p* = 0.148; Saline vs. Saline and Cold Exposure *p* = 0.247
Central (25–35 min)	67.40 ± 12.43	50.20 ± 6.17	22.30 ± 3.41	F(2,17) = 12.84, η^2^ = 0.60	<0.001	Saline vs. 5′-AMP and Cold Exposure *p* < 0.001 *; Saline and Cold Exposure vs. 5′-AMP and Cold Exposure *p* = 0.021 *; Saline vs. Saline and Cold Exposure *p* = 0.272
Final (50–60 min)				H(2) = 5.22, ε^2^ = 0.19	0.074	-
**C. External (peripheral) crossings**						
**Time Block**	**Saline**	**Saline and Cold Exposure**	**5′-AMP and Cold Exposure**	**Test Statistic**	***p***	**Post Hoc**
Central (25–35 min)	173.40 ± 31.47	141.60 ± 16.33	73.70 ± 9.29	F(2,17) = 9.72, η^2^ = 0.53	0.002	Saline vs. 5′-AMP and Cold Exposure *p* = 0.002 *; Saline and Cold Exposure vs. 5′-AMP and Cold Exposure *p* = 0.030 *; Saline vs. Saline and Cold Exposure *p* = 0.502
Final (50–60 min)				H(2) = 5.87, ε^2^ = 0.23	0.053	-

ANOVA with Tukey’s HSD post hoc test when assumptions met; Kruskal–Wallis H test with Bonferroni-corrected Mann–Whitney U test otherwise. * *p* < 0.05.

**Table 3:** Ethogram behavioral frequency and duration across treatment groups. Values represent mean ± SEM. Group sizes and statistical framework as in Table 2. Both full-session (60 min) and sub-period results are reported; empty cells indicate values not individually listed. Duration values are the cumulative time spent in each behavior summed across the period, not per-bout durations. Several control means in the final period are close to zero, so ratios between groups in that period are unstable; absolute values should be used for comparison. * *p* < 0.05.

**A. Frequency (bouts)**							
**Behavior**	**Period**	**Saline**	**Saline and Cold ** ** Exposure**	**5′-AMP and Cold ** ** Exposure**	**Test Statistic**	***p***	**Post Hoc**
Vertical exploring	Full	380.00 ± 45.80	293.00 ± 57.70	183.30 ± 41.60	F(2,17) = 4.38, η^2^ = 0.34	0.029	Saline vs. 5′-AMP and Cold Exposure *p* = 0.027 *; Saline vs. Saline and Cold Exposure *p* = 0.525; Saline and Cold Exposure vs. 5′-AMP and Cold Exposure *p* = 0.270
Vertical exploring	Initial (0–10)	139.40 ± 18.28	86.20 ± 23.14	58.20 ± 12.16	F(2,17) = 6.09, η^2^ = 0.42	0.010	Saline vs. 5′-AMP and Cold Exposure *p* = 0.008 *; Saline vs. Saline and Cold Exposure *p* = 0.148; Saline and Cold Exposure vs. 5′-AMP and Cold Exposure *p* = 0.467
General exploring	Full	358.60 ± 41.02	289.20 ± 50.14	201.70 ± 32.47	F(2,17) = 4.12, η^2^ = 0.33	0.035	Saline vs. 5′-AMP and Cold Exposure *p* = 0.032 *; Saline vs. Saline and Cold Exposure *p* = 0.545; Saline and Cold Exposure vs. 5′-AMP and Cold Exposure *p* = 0.290
General exploring	Initial (0–10)	138.40 ± 14.83	94.00 ± 23.24	70.20 ± 9.28	F(2,17) = 5.74, η^2^ = 0.40	0.012	Saline vs. 5′-AMP and Cold Exposure *p* = 0.009 *; Saline vs. Saline and Cold Exposure *p* = 0.166; Saline and Cold Exposure vs. 5′-AMP and Cold Exposure *p* = 0.479
Self-grooming	Full	12.60 ± 4.12	10.20 ± 1.62	24.70 ± 5.58	F(2,17) = 2.39, η^2^ = 0.22	0.122	-
Face washing	Full				F(2,17) = 1.43, η^2^ = 0.14	0.267	-
Inactivity	Full	8.00 ± 3.39	7.00 ± 1.76	25.20 ± 6.29	F(2,17) = 3.46, η^2^ = 0.29	0.055	-
Inactivity	Final (50–60)	3.60 ± 1.50	0.20 ± 0.20	7.30 ± 1.80	H(2) = 7.85, ε^2^ = 0.34	0.020	Saline and Cold Exposure vs. 5′-AMP and Cold Exposure *p* = 0.031 *
Jumping	Full				H(2) = 1.17, ε^2^ = 0.00	0.558	-
**B. Cumulative duration (total seconds per period)**							
**Behavior**	**Period**	**Saline**	**Saline and Cold Exposure**	**5′-AMP and Cold ** ** Exposure**	**Test Statistic**	***p***	**Post Hoc**
Vertical exploring	Full	721.39 ± 43.64	634.59 ± 122.50	433.61 ± 91.42	F(2,17) = 2.47, η^2^ = 0.23	0.114	-
Vertical exploring	Initial (0–10)	152.69 ± 14.59		78.79 ± 15.17	F(2,17) = 4.00, η^2^ = 0.32	0.038	Saline vs. 5′-AMP and Cold Exposure *p* = 0.030 *; Saline vs. Saline and Cold Exposure *p* = 0.186; Saline and Cold Exposure vs. 5′-AMP and Cold Exposure *p* = 0.774
General exploring	Full	1005.33 ± 71.00	1062.17 ± 89.41	976.94 ± 59.80	F(2,17) = 0.35, η^2^ = 0.04	0.707	-
Self-grooming	Full	47.62 ± 24.71	50.77 ± 26.81	184.39 ± 55.60	H(2) = 4.27, ε^2^ = 0.13	0.118	-
Self-grooming	Final (50–60)		15.45 ± 8.37	88.24 ± 29.18	H(2) = 6.75, ε^2^ = 0.28	0.034	Saline and Cold Exposure vs. 5′-AMP and Cold Exposure *p* = 0.058 (marginally non-significant after Bonferroni)
Face washing	Full				F(2,17) = 2.76, η^2^ = 0.25	0.092	-
Inactivity	Full	29.42 ± 19.63	27.17 ± 15.58	136.96 ± 51.36	H(2) = 2.41, ε^2^ = 0.02	0.300	-
Inactivity	Final (50–60)	23.86 ± 18.25	0.86 ± 0.86	38.76 ± 13.59	H(2) = 6.37, ε^2^ = 0.26	0.041	Saline and Cold Exposure vs. 5′-AMP and Cold Exposure *p* = 0.045 *
Jumping	Full				H(2) = 1.17, ε^2^ = 0.00	0.558	-

Statistical framework as in Table 2. Empty cells: values not individually reported. * *p* < 0.05.

**Table 4:** Y-maze spontaneous alternation performance. Data represent mean ± SEM. Group sizes as in Table 2. One-way ANOVA was applied after confirming normality and equal variances. Each test statistic is followed by its effect size (η^2^). All groups performed above the 50% chance level, and no between-group difference in spontaneous alternation was detected.

Measure	Saline	Saline and Cold Exposure	5′-AMP and Cold Exposure	Test Statistic	***p***
Total arm entries	49.20 ± 6.70	38.00 ± 2.35	35.60 ± 4.46	F(2,17) = 1.93, η^2^ = 0.19	0.176
Spontaneous alternation (%)	54.75 ± 4.23	57.62 ± 4.85	59.10 ± 5.54	F(2,17) = 0.15, η^2^ = 0.02	0.863

One-way ANOVA after confirming normality and equal variances. All groups above 50% chance level.

**Table 5:** Behavioral latency to first occurrence analyzed using survival methods. Median latency (seconds) with penetrance (events/total) is shown below each value. Observation window: 600 s. Group differences were assessed by log-rank test; Cox proportional hazards regression estimated hazard ratios (HR) with 95% CI relative to Saline controls. An HR below 1 indicates delayed initiation; values above 1 indicate accelerated onset. “Not reached” means fewer than half of the animals exhibited the behavior within the observation window. This approach follows recommendations by Jahn-Eimermacher et al. [43]. * *p* < 0.05.

Behavior	Saline Median (s)	Saline and Cold Exposure Median (s)	5′-AMP and Cold Exposure Median (s)	Log-Rank chi-sq(2)	***p***	HR [95% CI] 5′-AMP and Cold Exposure vs. Saline	HR [95% CI] Saline and Cold Exposure vs. Saline
Wall rearing	19.6 (5/5)	23.9 (5/5)	24.4 (10/10)		0.385		
Rearing	86.0 (5/5)	256.1 (5/5)	201.9 (10/10)	7.65	0.022 *	0.23 [0.07, 0.79] *p* = 0.020 *	0.18 [0.04, 0.81] *p* = 0.025 *
Self-grooming	296.4 (4/5)	not reached (3/5)	198.6 (10/10)	5.17	0.076		
Inactivity	not reached (2/5)	284.0 (5/5)	141.0 (7/10)	3.85	0.146	3.86 [0.79, 18.81] *p* = 0.095	4.42 [0.82, 23.83] *p* = 0.084
Face washing	241.0 (5/5)	152.0 (5/5)	197.4 (10/10)		0.474		

Pairwise log-rank: Rearing Saline vs. 5′-AMP and Cold Exposure *p* = 0.014 *; Self-grooming Saline and Cold Exposure vs. 5′-AMP and Cold Exposure *p* = 0.029 *; Inactivity Saline vs. Saline and Cold Exposure *p* = 0.006 *. HR from Cox regression, Saline as reference. HR < 1 = delayed; HR > 1 = accelerated. “Not reached” =<50% exhibited behavior. * *p* < 0.05.

## Data Availability

The datasets generated and analyzed during the current study are available from the corresponding author upon reasonable request.
